# Lipome géant du dos: rapport de cas avec revue de la littérature

**DOI:** 10.11604/pamj.2022.42.292.21047

**Published:** 2022-08-18

**Authors:** Abdou Niasse, Papa Mamadou Faye, Abdourahmane Ndong, Ousmane Thiam, Ousmane Gueye, Mouhamadou Lamine Gueye, Ibrahima Sitor Souleymane Sarr, Yacine Seye, Alpha Oumar Toure, Mamadou Seck, Mamadou Cisse, Madieng Dieng

**Affiliations:** 1 Service de Chirurgie Générale, Centre Hospitalier Universitaire Aristide Le Dantec, Dakar, Sénégal,; 2 Service de Chirurgie Générale, Hôpital Dalal Jamm, Dakar, Sénégal,; 3 Service de Chirurgie Générale, Hôpital Matlaboul Fawzaïni de Touba, Diourbel, Sénégal

**Keywords:** Lipome géant, liposarcome, lipomectomie, cas clinique, Giant lipoma, liposarcoma, lipomectomy, case report

## Abstract

Le lipome est une tumeur bénigne des parties molles. Il s´agit d´une prolifération bénigne d´adipocytes matures. Il est qualifié de géant lorsque son poids dépasse 1 kg ou son diamètre supérieur à 5 cm. La gêne fonctionnelle et esthétique peut être un motif principal d´exérèse chirurgicale. Ils peuvent se localiser partout, mais surtout au niveau de la face postérieure du thorax. Nous rapportons un cas de lipome géant de la face postéro-supérieure gauche du thorax.

## Abstract (en)

Lipoma is a benign soft tissue tumour. It is a benign proliferation of mature adipocytes. It is described as giant when its weight exceeds 1 kg or its diameter exceeds 5 cm. Functional and aesthetic impairment may be a major reason for surgical excision. It can be located everywhere, but it mainly occurs in the posterior segment of the chest. We here report a case of giant lipoma of the left posterior-superior segment of the chest.

## Introduction

Les lipomes sont des tumeurs bénignes qui se développent dans les zones où le tissu adipeux est abondant [1]. Le lipome est qualifié de « géant » quand la pièce d´exérèse dépasse 5 cm de diamètre [2]. Les lipomes superficiels sont le plus souvent localisés au dos, aux épaules, au cou et à l´abdomen, suivis des bras et des cuisses [3]. Nous rapportons un cas de lipome géant de la face postéro-supérieure gauche du thorax.

## Patient et observation

**Information du patient:** il s´agissait d´un patient âgé de 43 ans, sans antécédents pathologiques particuliers. Le patient présentait une masse de la face postéro-supérieure gauche du thorax. Cette masse évoluait depuis sept ans. Elle était indolore et rendait difficile le décubitus dorsal et pesant sur le dos. Ceci avait motivé un traitement traditionnel par massage et phytothérapie sans succès.

**Figure 1:**
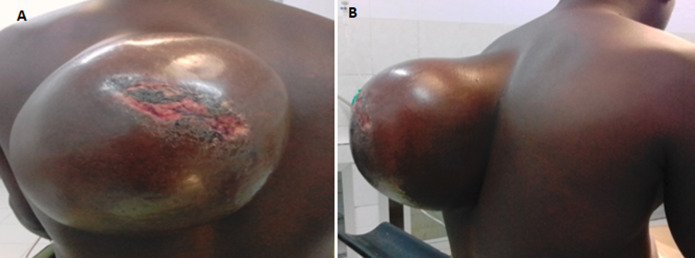
lipome, A) vue de face ; B) vue de profil

**Résultats cliniques:** à l´examen clinique, il présentait une tumeur mobile par rapport à la peau et à la paroi thoracique d´environ 23 cm/15 cm de grand axe, de consistance molle, siégeant à la face postéro-supérieure gauche du thorax. Elle était indolore avec une ulcération de 5 cm à son sommet sans vascularisation visible (Figure 1).

**Figure 2:**
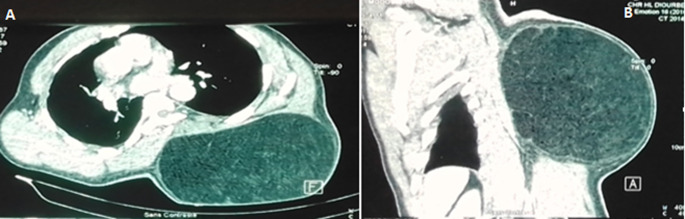
images scannographiques du lipome, A)coupe sagittale; B) coupe transversale

**Démarche diagnostique:** la tomodensitométrie thoracique réalisée avait montré une volumineuse masse oblongue sous cutanée dorsale gauche de densité graisseuse (-100 UH) multi loculée mesurant 22,94 cm/15,87 cm, à contours bien limités, réguliers. Sa paroi n´était pas rehaussée par le produit de contraste (Figure 2).

**Figure 3:**
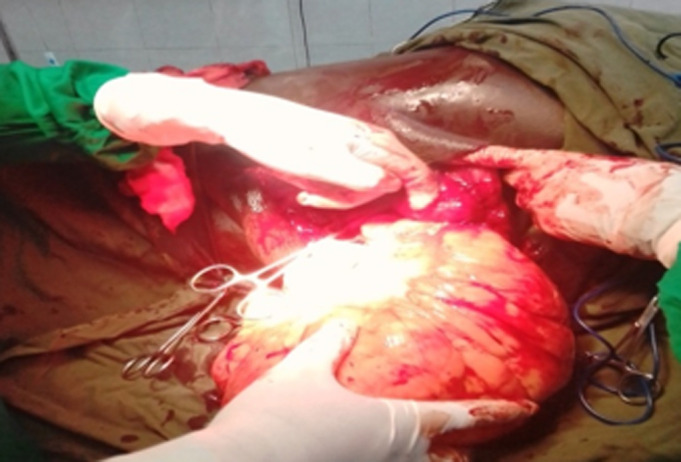
énucléation du lipome (base d’implantation du lipome sur le thorax)

**Figure 4:**
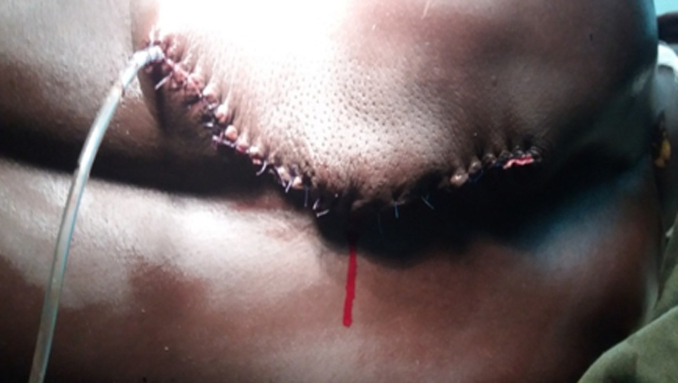
plaie opératoire

**Figure 5:**
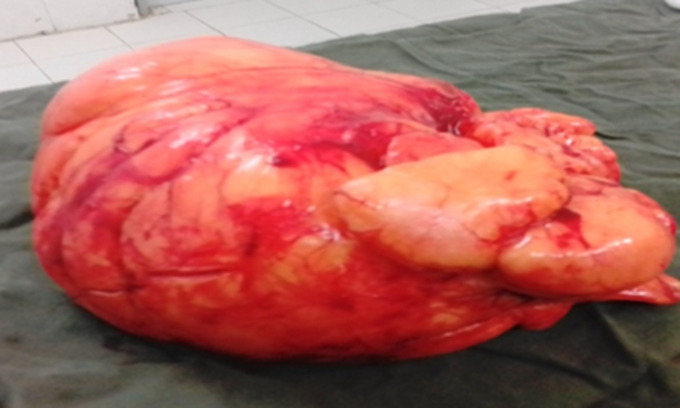
pièce opératoire

**Intervention thérapeutique et suivi:** une exérèse totale de la tumeur a été réalisée par incision arciforme, puis décollement antérieur et postérieur aux doigts et énucléation après ligature du pédicule nourricier (Figure 3). L´excédent de peau était réséqué permettant une fermeture esthétique sur drain de Redon aspiratif (Figure 4). La pièce d´exérèse mesurait 23 cm/15 cm et pesait 2800 grammes (Figure 5). Les suites opératoires étaient simples avec ablation du drain au deuxième jour post-opératoire. L´étude histologique de la pièce opératoire avait conclu à un lipome lipocytique sans signes de malignité. Après un recul de six mois, le patient ne présentait pas de récidive.

**Consentement du patient:** le patient a donné son consentement éclairé pour la publication de ce cas.

## Discussion

Le lipome superficiel sous-cutané est très fréquent, il représente 16 à 50% de toutes les tumeurs des tissus mous [4]. Les patients sont typiquement d´âge moyen, sans prédominance de sexe lorsque le lipome est solitaire [4,5]. Ils peuvent se localiser n´importe où dans le corps, avec environ 15 à 20% situés au niveau du dos [3]. L´étiopathogénie des lipomes est mal connue [5]. Ils peuvent être dus à des traumatismes minimes et répétés [5]. Dans 5 à 15% des cas, les lipomes sont multiples. Il existe alors une prédominance masculine avec, dans un tiers des cas, une transmission héréditaire, habituellement autosomique dominante (lipomes multiples familiaux) [4,5]. Ces lipomes apparaissent en général entre 30 et 50 ans dans le tissu sous-cutané des membres et du tronc, épargnant souvent le cou et les épaules. D´autres impliquent également l´obésité sans preuve formelle [4].

Leur nombre est très variable. Ils ne dégénèrent pas lorsque l´exérèse est totale jusqu´en zones saines. Ce qui est parfois difficile pour les lipomes profonds [5]. Les lipomes sont fréquemment asymptomatiques comme chez notre patient. On peut noter de vagues douleurs, une tension ou une compression d´un nerf périphérique [4]. La plainte est essentiellement esthétique puisqu´ils sont massifs et disgracieux. Mais la gêne fonctionnelle comme chez notre patient est loin d´être négligeable, car ils peuvent être à l´origine de macérations cutanées, de compression nerveuse et d´attitude vicieuse de la colonne vertébrale. Le diagnostic est orienté par l´examen clinique. Le lipome est souvent indolore et se traduit habituellement par une tumeur molle, régulière et mobile. Posch a décrit le test clinique d´application de glace sur la tumeur, qui en cas de lipome entraîne une solidification de la masse [6]. L´évolution habituelle est une croissance lente, qui peut se stabiliser spontanément [6]. La plupart des lipomes sont de petite taille, 80% mesurant moins de 5 cm, le reste faisant typiquement moins de 10 cm et rarement plus [6].

L´ imagerie par résonance magnétique (IRM) est l´examen d´imagerie de référence dans l´exploration des tumeurs des parties molles du fait de sa haute sensibilité [2]. Elle précise la nature de la lésion, son extension locale et ses rapports avec les éléments vasculo-nerveux. Néanmoins, la tomodensitométrie peut également aider au diagnostic de lipome [2]. Au scanner et en IRM, le lipome est caractérisé par une densité entre -65 et -120 UH et un signal identique à celui de la graisse sous-cutanée. Il est habituellement limité par une fine capsule fibreuse hypointense et hypodense. Cette capsule est observée en IRM dans près de la moitié des lipomes superficiels et peut mesurer jusqu´à 3 mm d´épaisseur [5]. Dans l´autre moitié, le lipome se fond de manière progressive avec la graisse sous-cutanée adjacente, sans masse individualisable. Un marqueur permettant de repérer la tuméfaction est alors utile, de même qu´une comparaison avec le côté controlatéral [5]. Le lipome peut contenir de fins septums internes (< 2 mm d´épaisseur), ne prenant pas ou peu le contraste. La capsule peut légèrement se rehausser après injection [7,8].

Le diagnostic différentiel se pose avec d´autres tumeurs des tissus mous tels que les kystes ganglionnaires, les tumeurs à cellules géantes, les myxomes, les angiolipomes, le lipofibrome intraneural et le liposarcome [4]. Le Tableau 1 [5] récapitule la différence entre liposarcome et lipome [9]. Le traitement chirurgical est de référence précédée par des biopsies afin de confirmer le diagnostic et autoriser alors l´exérèse et l´usage de techniques de conservation cutanée pour recouvrir toute perte de substance résiduelle. Cette démarche thérapeutique est souvent difficile dans notre contexte où l´IRM et l´histologie ne sont pas toujours disponibles en zone rurale. Nous ne disposons que de l´exérèse première et une étude histologique de la pièce opératoire si l´examen clinique et l´imagerie orientent vers un lipome bénin. Certains auteurs optent pour une lipoaspiration première afin de réduire le volume tumoral [7]. Le résultat esthétique serait meilleur et la morbidité postopératoire moindre [7]. Aux membres, le taux de récidive des lipomes superficiels est de 1%, celui des lipomes intramusculaires et du dos est de 12% [10].

**Tableau 1:** principaux éléments différentiels entre lipomes et liposarcomes [5]

	Lipome superficiel	liposarcome
Topographie	Graisse sous-cutanée dos, épaule, cou, abdomen, bras, cuisses	Muscles et fascias cuisse, rétro péritoine
Taille	< 5 cm (80 %) < 10 cm (95 %)	Exceptionnellement >5 cm
contours	Nets le plus souvent	Nets le plus souvent
Forme	Unilobulaire	Plurilobulaire (> 90 %)
Cloisons épaisses	Rares (fibrolipomes)	Présentes et vascularisées
Nodules	Absents	Présents

## Conclusion

Bien qu´il soit de nature bénigne, le lipome géant est souvent source de gêne fonctionnelle importante. Son traitement est chirurgical. La hantise du chirurgien reste la dégénérescence maligne. Dans nos contextes où l´histologie n´est pas toujours disponible, nous recommandons une chirurgie d´exérèse première en cas de forte suspicion de lipome, avec étude anatomopathologique systématique de la pièce opératoire.
